# Efficacy of a Conservative Weight Loss Program in the Long-Term Management of Chronic Upper Airway Obstruction

**DOI:** 10.1155/2009/396523

**Published:** 2009-08-18

**Authors:** Ryan C. Case, John Schweinfurth

**Affiliations:** Department of Otolaryngology, The University of Mississippi Medical Center, Jackson, MS 39216, USA

## Abstract

*Objective*. Obesity is a significant contributor to oxygen demand and dynamic airway obstruction. The objective of the current study is to determine the long-term success of conservative measures directed toward weight reduction on airway management without respect to specific airway disease etiology. *Methods*. Patients with chronic airway obstruction secondary anatomic lesions or obstructive sleep apnea were recruited and followed prospectively. Demographics, initial and final weights, diagnosis, and followup information were recorded. Patients were referred to a registered dietician, provided counseling, and started on a weight-loss regimen. Outcome measures were change in body mass index (BMI) and rate of decannulation from weight loss alone. *Results*. Of fourteen patients, ten remained tracheostomy-dependent and four had high-grade lesions with the potential for improvement in oxygen demand and dynamic airway collapse with weight loss. The mean follow up period was 25 months. The mean change in BMI was an increase of 1.4 kg/m^2^ per patient. *Conclusions*. Conservative measures alone were not effective in achieving weight reduction in the population studied. This may be due to comorbid disease and poor compliance. The promise of decannulation was an insufficient independent motivator for weight loss in this study. Although the theoretical benefits of weight loss support its continued recommendation, the long-term success rate of conservative measures is low. More aggressive facilitated interventions including pharmacotherapy or bariatric surgery should be considered early in the course of treating airway disease complicated by obesity.

## 1. Introduction

The benefits for weight loss in the obese patient are numerous. In today's society, laymen are aware of the health benefits to weight reduction that essentially affect every system of the body. Many of the benefits such as lower blood pressure, better lipid profiles, and glucose control have a profound impact on long-term health, but the patients may not feel the benefit in the short-term and thus revert to their previous lifestyle after having no apparent immediate benefit from weight loss. Studies have shown that weight loss has beneficial effects on clinical status in obese patients with airway disorders that is obvious to the patient such as decreased dyspnea and increased exercise tolerance [1, 2]. The question remains as to whether the immediate benefits of weight loss in obese patients with airway disorders will provide the motivation for that population as a whole to lose weight.

An association between obesity and chronic respiratory disease is becoming more apparent as the incidence of obesity is increasing. Most respiratory and airway disorders have been shown to be affected in some way by obesity [3, 4]. For example, in asthma and chronic bronchitis, excess weight is thought to contribute through a decrease in pulmonary compliance as well as through the production of inflammatory mediators [3, 4]. Excess weight contributes to upper airway obstruction both through an increase in pharyngeal mass, narrowing of the upper airways, and increased work of breathing and oxygen demand [5].

It has been established that obesity increases oxygen demand during exercise. Recent studies have further shown that obesity also increases oxygen consumption (V_O2_) at rest [6]. The mechanism is thought to be the increased circulatory burden posed by excess adipose tissue. The obese patient with upper airway disease is, therefore, at greater risk for respiratory decompensation [6]. It has also been shown that obese individuals have a lower anerobic threshold further suggesting an increased V_O2_ [5]. Several studies have demonstrated that weight loss is efficacious in improving symptoms in patients with respiratory diseases [1]. For patients with an anatomic airway obstruction, this occurs in part through decreased systemic oxygen demand. The amelioration of comorbidities such as diabetes and hypertension is also likely to contribute positively.

Effective weight loss is difficult for many patients. The social and psychological aspects of obesity are complex and many patients are unable to achieve long-term weight management [7]. No available studies determine if weight loss can improve respiratory symptoms from anatomic airway obstruction. The present study intends to determine if properly counseled obese patients with upper airway obstruction were compliant and successful with a prescribed weight-loss regimen.

## 2. Methods

Institutional review board approval was obtained. Patients with a body mass index (BMI) greater than 30 with anatomic airway obstruction who were currently tracheostomy-dependent or requiring tracheostomy pending weight loss were selected for study. Obese patients with airway disease received regular followup for weight measurement as well as dietary and diabetic education referrals for weight management counseling. Dietary recommendations reflected the American Diabetic Association guidelines which consisted of 1500 kcal for women and 1800 kcal for men including low-fat principles and portion control. Patients were given an exercise regimen that included a minimum of 10 minutes of moderate activity daily. Patients with etiologies of airway obstruction attributable to vocal cord paralysis and subglottic stenosis received surgical intervention for their airway obstruction consisting of unilateral cordotomy or lysis of stenosis. Patients were followed at regular intervals and weights documented. Data recorded for each patient included age, sex, BMI at the time of tracheostomy, reason for tracheostomy, and changes in BMI. Initial and final study BMI means were compared for statistical analysis using a paired *t*-test. Candidates for decannulation were downsized to a #4 Shiley (Tyco Inc., Pleasanton, Calif, USA), and underwent capping trials for 14 days before tracheostomy tube removal.

## 3. Results

A total of 15 patients, 5 male and 10 female, were recruited for study, of which 13 had sufficient data available for study. Demographic data, airway disease, and outcomes are summarized in Table 1. Patient age ranges from 26 to 58 years old, with a mean age of 44.5 years. None of our patients had insulin-dependant diabetes mellitus, cardiogenic fluid retention, or renal insufficiency. The patients were followed over an average of 25 months, with a range of 6 to 48 months. The mean initial BMI for the study group was 43 kg/m^2^ and the final 44.4 kg/m^2^ for an overall gain of 1.4 kg/m^2^. This was not statistically significant at *P* = .32. The data for men and women was similar at 43.6/43.3 and 42.7/44.7 initial/final BMI, respectively. We defined significant weight loss as a decrease in weight of 5% over thirty days. During the study, only two patients experienced significant weight loss (45, 60 lbs) secondary to gastrointestinal illnesses unrelated to the weight loss measures and were subsequently successfully decannulated. Two patients in the study are near decannulation due to improving clinical status after weight loss.

## 4. Discussion

Obesity contributes to respiratory pathology in several ways in the overweight patient. Anatomically, increased soft tissue mass contributes to upper airway obstruction as in the patient suffering from sleep apnea or by complicating the course of a patient with a pre-existing airway stenosis [2, 8]. Decreased chest wall compliance also negatively impacts respiration, and obese individuals are known to have decrease functional residual capacity and expiratory reserve volume which improves with weight loss [2, 5].

Once thought to be a relatively inert tissue, adipose tissue is now the subject of much study into its role as a metabolically active tissue. It is now known that adipose tissue has multiple systemic deleterious effects and complicates chronic diseases such as diabetes mellitus, hypertension, and dyslipidemia, and it most recently has been suggested to exacerbate respiratory diseases through the production of inflammatory mediators [3, 4]. Obesity is also thought to contribute to bronchial hyperresponsiveness through the exacerabation of gastroespohageal reflux, a known risk factor for asthma [3].

For many patients who are both obese and suffer from airway obstruction, excess adipose tissue may be creating a metabolic profile that aggravates their respiratory disease. Obese patients have a higher peripheral oxygen uptake than their lean counterparts that cannot all be attributed to the increase in work of breathing [5]. This increased oxygen demand is likely due to the metabolic activity of adipose cells, essentially stealing oxygen from skeletal muscles during exercise and resulting in a low anerobic threshold.

Extra adipose tissue can also cause difficulties for patients in that the extratissue creates increased work that the musculoskeletal system must then overcome. The added demand on muscles that are already relatively oxygen-deprived likely contributes to the dyspnea felt by obese patients during exertion. Maniscalco et al. quantified increased performance and decreased dyspnea in overweight patients who lost weight following bariatric surgery [2]. The combined effect of these elements, the mechanical disruption of respiration, production of inflammatory mediators, burden on the musculoskeletal system, and increase in oxygen demand likely have a synergistic effect on the obese with an airway obstruction. For many of the patients studied, airway dysfunction is likely not due to visceral fat accumulation as several patients continued to progress to a point where tracheostomy was required. Obesity remains a detriment to respiratory function: decreased chest wall compliance, increased work load, increased oxygen demand, and possible metabolic effects.

The management of anatomic airway obstruction and obesity is complex. The surgical treatment of bilateral vocal cord paralysis, for example, is an ongoing compromise of respiration, voice, and deglutition. Once the optimum glottic airway with respect to these functions has been achieved, other than permanent tracheostomy, significant weight loss is the only remaining option for many patients. Despite the availability of definitive airway interventions, it is likely that many obese patients would exhibit symptomatic dyspnea even with an anatomically normal airway. Unfortunately for many patients with upper airway obstruction, long-term weight loss is not achieved despite the promise of improved respiratory function which has a significant impact on quality of life and other comorbid diseases.

The current study is not intended as a clinical trial of different treatments for airway disorders but simply to provide “real-world” outcome information on conservative measures for weight loss in a small, airway-diseased population. A common situation that occurs is weighing between difficult surgical options that carry significant risk of failure; risk that could be offset by significant weight reduction. However, despite the obvious motivating factor of ending dependence on an indwelling tracheostomy, significant weight loss was not achieved by the group as a whole using conservative measures without pharmacotherapy. In the majority of cases, this was due to noncompliance with the dietary and exercise recommendations. In a large population-based study, significant long-term weight loss was achieved in only 28% of patients receiving intense multidisciplinary intervention for their obesity [7]. Despite the many ways that obesity complicates airway disorders, improvement of respiratory and ventilatory function and the resulting presumed positive impact on quality of life from weight loss did not provide significant motivation for weight loss in our patient population. Clearly, if significant weight loss is to be achieved, more aggressive measures, such as bariatric surgery, must be seriously considered.

Patients with etiologies of airway obstruction attributable to vocal cord paralysis and subglottic stenosis received appropriate surgical intervention for their airway obstruction, but still did not achieve decannulation. This failure was attributed to continued obesity.

## 5. Conclusion

The challenges for clinicians and patients who struggle with obesity are many: the disease touches nearly every aspect of the patient's life and is certainly one of the most difficult conditions to treat. Social and economic limitations likely conspire to complicate weight loss, and even when patients have all available resources and support at hand, long-term results are still difficult to achieve. At the end, it may prove difficult for medicine to address a disease whose etiology is more cultural than physiological. Therefore, sustained interventions at multiple levels are likely required if weight loss is to be an important part of airway management.

## Figures and Tables

**Table 1:** Demographics and weight loss outcomes.

Age	Sex	Number of Visits	Initial BMI	Final BMI	Δ BMI	Etiology of airway obstruction
46	Male	6	42.9	39.7	−3.2	OSA
56	Female	32	43.9	43.7	−0.2	Subglottic stenosis, trachmalacia
45	Female	19	28	34.3	6.3	BTVC paralysis
36	Male	14	57.9	71.6	13.7	OSA, laryngomalacia
26	Female	9	43.7	44.2	0.5	Subglottic stenosis
44*	Male	16	40	39.9	−0.1	BTVC paralysis
32*	Female	7	48.3	40.3	−8	OSA, tracheomalacia
27	Male	9	32.2	27.5	−4.7	BTVC paralysis, OSA
55	Male	10	44.8	43.3	−1.5	OSA
54	Female	6	47.2	53.2	6	Subglottic stenosis
58	Female	16	38.4	37.8	−0.6	Subglottic stenosis
39	Female	12	42.6	47.1	4.5	BTVC paralysis
57	Female	9	42.9	52.4	9.5	OSA

*Patient was decannulated after weight loss attributable to an unrelated medical illness.
